# Modern Innovative Solutions in Improving Outcomes in Chronic Obstructive Pulmonary Disease (MISSION COPD): Mixed Methods Evaluation of a Novel Integrated Care Clinic

**DOI:** 10.2196/ijmr.9637

**Published:** 2019-10-01

**Authors:** Eleanor Lanning, Jayne Longstaff, Thomas Jones, Claire Roberts, Daniel Neville, Ruth DeVos, Will Storrar, Ben Green, Thomas Brown, Anthony Leung, Carole Fogg, Rachel Dominey, Paul Bassett, Paul Meredith, Anoop J Chauhan

**Affiliations:** 1 Portsmouth Hospitals NHS Trust Portsmouth United Kingdom; 2 Badgerswood Surgery Borden United Kingdom; 3 University of Portsmouth Portsmouth United Kingdom; 4 Wessex Academic Health Science Network Southampton United Kingdom; 5 Stats Consultancy Berkshire United Kingdom

**Keywords:** chronic obstructive pulmonary disease, comorbidity, multidisciplinary, delivery of health care, diagnosis

## Abstract

**Background:**

Chronic obstructive pulmonary disease (COPD) is the second-leading cause of death in the United Kingdom and accounts for 1.7% of bed days in acute hospitals. An estimated two-third of patients with COPD remain undiagnosed.

**Objective:**

Modern Innovative Solutions in Improving Outcomes in Chronic Obstructive Pulmonary Disease (MISSION COPD) aimed to proactively identify patients from primary care who were undiagnosed or had uncontrolled COPD and to provide a comprehensive integrated multidisciplinary clinic to address the needs of this complex group for improving diagnosis, personalizing therapy, and empowering patients to self-manage their condition.

**Methods:**

This clinic was led by a respiratory specialist team from Portsmouth Hospitals NHS Trust working with five primary care surgeries in Wessex. A total of 108 patients were reviewed, with 98 patients consenting to provide additional data for research. Diagnoses were changed in 14 patients, and 32 new diagnoses were made.

**Results:**

Reductions were seen across all aspects of unscheduled care as compared to the prior 12 months, including in emergency general practitioner visits (3.37-0.79 visits per patient, *P*<.001), exacerbations (2.64-0.56 per patient, *P*=.01), out-of-hours calls (0.16-0.05 per patient, *P*=.42), and hospital admissions (0.49-0.12 per patient, *P*=.48). Improvements were observed in the quality of life and symptom scores in addition to patient activation and patient-reported confidence levels.

**Conclusions:**

This pilot demonstrates that the MISSION model may be an effective way to provide comprehensive gold-standard care that is valued by patients and to promote integration across sectors.

## Introduction

Over 1 million people in the United Kingdom suffer from chronic obstructive pulmonary disease (COPD), which remains the second-leading cause of death in the United Kingdom. These people place a significant burden on the National Health Service through urgent unscheduled care visits in primary and secondary care. However, there are a significant number of patients who remain undiagnosed despite the presence of symptoms or risk factors that are often not recognized, and the opportunity to diagnose COPD is often missed [1]. In people who are diagnosed, management within primary care is led by nursing staff and is protocol driven with Quality Outcomes Framework prompts [2], resulting in yearly reviews that are largely disease focused. This approach risks underidentifying comorbidities as well as the psychological and social impacts of COPD. Diagnosis is often challenging: One meta-analysis of a variety of case-finding strategies reported a COPD diagnostic yield of 1.7%-30.5%, with one study finding that 37.2% of new diagnoses were severe when using a spirometric definition [3].

A Cochrane review identified 25 randomized controlled trials of integrated disease management interventions in COPD and concluded that such management improved the quality of life and reduced acute hospital admissions [4]. The review identified over 10 important aspects (eg, education, self-management, nutrition, exercise, and case management), but no single integrated clinic delivered the full suite of these holistic interventions in patients diagnosed with COPD, nor could the authors identify a comprehensive clinic targeting undiagnosed COPD.

We describe our experience of Modern Innovative Solutions in Improving Outcomes in Chronic Obstructive Pulmonary Disease (MISSION COPD), a novel model of integrated care for airways disease built on the success of the preceding MISSION Asthma [5] model. We aimed to proactively identify patients from electronic primary care records with either poorly controlled disease or those in whom history was suggestive of diagnosis but who had until then remained undiagnosed. Each patient underwent a streamlined series of holistic assessments (delivered by both primary and secondary care teams) incorporating smoking cessation advice; a medical review including a comprehensive comorbidity screen; specialist physiological investigations including fractional exhaled nitric oxide, an individualized inhaler technique review, a personalized self-management plan, and written literature to support and encourage self-management; and a chance to participate in research. Group education sessions were delivered on the day of the clinic, and further sessions were delivered 3 months later. Clinics were held during weekends to increase accessibility to the working population. By combining both primary and secondary expertise in the patient with a COPD pathway, true vertical integration of care was demonstrated.

This paper describes the implementation of the MISSION COPD pilot project and the patients who accessed the service and summarizes the impact on both patient and health service outcomes.

## Methods

### Objectives

This research study accompanied the MISSION COPD Quality Improvement Programme. The primary objective of the research was to assess whether the number COPD exacerbations (prednisolone or equivalent≥30 mg for >3 days or antibiotics for >3 days [6]) improved the condition of the MISSION patients during the 6 months after the clinic compared with the 12 months before the clinic and to assess whether hospital admissions changed during the 6 months after the clinic.

The secondary objectives for participants with an established diagnosis of COPD were to assess (1) whether the number of nonelective general practitioner (GP) visits for COPD changed during the 6 months after the clinic; (2) the severity of COPD in the MISSION clinics; (3) the number of COPD exacerbations in 6 months pre-MISSION and post-MISSION; (4) the patient activation measure (PAM) pre-MISSION and post-MISSION; (5) the frequency and severity of comorbidities in the COPD population; (6) COPD control using the COPD Assessment Test (CAT) questionnaire at 3 and 6 months; (7) inhaler technique (ie, correct technique or correct device); (8) lung function and phenotypes of patients with COPD seen in the MISSION clinic; (9) frequency of smoking; (10) the health economic impact of the MISSION service; (11) the number of patients with newly diagnosed COPD; and (12) lung function and phenotypes in patients newly diagnosed with COPD.

### Setting

Primary care–based clinics were performed between September and November 2015. Surgeries were drawn from 4 clinical commissioning groups’ footprints surrounding Queen Alexandra Hospital in Portsmouth. These clinical commissioning groups’ footprints are largely from rural or semirural settings. The surgery list sizes ranged from 6895 to 15,495 patients and the COPD list sizes ranged from 119 to 369.

This project was approved by the Berkshire Ethics Committee (approval number 15/NS/0085).

### Identification of Invitees for Clinics

Using the Grant Readiness Assessment Strategy Prep [7] COPD and case-finding tools, databases were searched using two sets of criteria. Criteria for patients with a suspected COPD diagnosis (case-finding cohort) were as follows: history of smoking; age≥35 years; presence of ≥3 respiratory symptoms coded in the last 24 months; prescription of an inhaler without a respiratory diagnosis; and prescription of ≥2 steroid or antibiotic courses in the prior 2 years for a chest complaint. Patients with an established COPD diagnosis but with uncontrolled COPD were defined as those having (1) moderate or higher Global Initiative for COPD stage; (2) body mass index<21 kg/m^2^; and (3) ≥2 contacts with the emergency department (ED) or out-of-hours services or ≥2 steroid courses in the prior 2 years. Referrals were also accepted from either cohort from the patient’s GP or practice nurse. We did not invite patients under active secondary care follow-up (defined as a visit for the same presentation within 6 months), but accepted patients previously known to receive secondary care and had been discharged. We also excluded patients who were housebound or had significant cognitive impairment, as the clinic structure was deemed unsuitable.

### Research Recruitment

All patients selected for clinic attendance were eligible to participate in the study if they were able to give informed consent. Patients were defined as being in a “care” cohort when a diagnosis of COPD was already known and in a “case-finding” cohort when a diagnosis was suspected.

### Modern Innovative Solutions in Improving Outcomes in Chronic Obstructive Pulmonary Disease Clinic

All participants were invited to a rapid COPD assessment clinic (RCAC) held within primary care. This carousel clinic encompassed targeted comorbidity screening questionnaires such as the Hospital Anxiety and Depression Score (HADS), Nijmegen, Epworth, Gastroesophageal Reflux Disease Questionnaire; physiological assessment of disease severity and phenotype (spirometry with reversibility and where appropriate, exhaled nitric oxide); smoking cessation; inhaler technique; and medical review. Small-group COPD education was delivered on the day, while the clinical team held a multidisciplinary discussion of each patient. The clinic session then ended with individualized feedback including a self-management plan for each participant.

Where the multidisciplinary team identified a need for further investigation, participants were invited to a one-day severe COPD–assessment clinic within 4 weeks of their RCAC appointment. Each patient underwent an individualized carousel of assessments and reviews that incorporated any number of physiotherapies, dietetics; symptoms control, psychology, social work, lung function laboratory assessments; echocardiographies; and computed tomography scans. All participants ended the clinic visit by being given further feedback and an update to their self-management plan where needed.

### Data Collection

Research participants were asked to complete clinical assessments and questionnaires at baseline, 3 months, and 6 months.

#### Capturing Outcomes Related to Unscheduled Care Usage

Data related to unscheduled care usage were collected from the primary care records (EMIS Web) for the 12 months prior to the first clinic review and for the 6 months afterward by a clinical fellow or experienced nurse. Unscheduled care usage included emergency GP appointments, out-of-hours GP contact, ED attendance, and hospital admissions where a respiratory symptom was a driver of the episode. The number of exacerbations was also documented, defined by a prescription of antibiotics and steroids for chest symptoms. Unscheduled GP visits were defined as any visit relating to the respiratory condition that was not a planned review or scheduled follow-up to a prior visit.

#### Disease Control and Severity

Baseline disease control was established using spirometry to measure the forced vital capacity and forced expiratory volume in 1 second, CAT [8], Medical Research Council score, St George’s Respiratory Questionnaire (SGRQ [9]) scores, and the Veterans Specific Activity Questionnaire scale scores. Changes in disease control were established by further CAT and SGRQ at 3 and 6 months.

#### Activation and Confidence

Patients were asked to rate their confidence in self-managing and their knowledge of COPD on first arrival at the RCAC by using a Likert scale. At 3 and 6 months, they were asked to rate, on a second scale, their agreement with the statement “Attending the MISSION COPD improved my confidence in managing my COPD.” PAM was used to measure activation at these points. If a person moves up one category of the PAM, it was considered a significant increase in activation [10].

#### Economic Evaluation

The costs of unscheduled care usage were derived from Personal Social Services Research Unit [11], and individuals’ use before and after the clinic was compared. The costs of clinic delivery were reported according to the team’s spending. Equivalent secondary care episode costs were calculated for investigations performed at the severe COPD clinic using current tariff rates at the host hospital. For the case-finding cohort, an additional preclinic cost of 3 GP visits was added as a typical diagnostic requirement (first review visit, spirometry visit, and follow-up).

### Statistical Analysis

The results were analyzed using Microsoft Excel (Microsoft Corp, Redmond, WA) and SPSS (version 23, Armonk, NY). The descriptive analysis of patient cohorts was guided by the data distribution or stated as a percentage of the cohort. Differences in unscheduled care usage were tested using Wilcoxon signed rank tests. Validated questionnaire outcomes were compared with the stated minimally important difference for each questionnaire before and after data were compared for each cohort and the total population, allowing the group to act as its own control.

## Results

### Delivery of Intervention

Clinics were held from September to November 2015, and 108 patients were assessed in the clinic, with 90 patients providing informed consent for research.

The baseline characteristics of the cohort are described in Table 1. The case cohort was younger and had fewer cumulative pack-years of smoking history. Lung function testing and dyspnea scores revealed greater severity of disease in the care cohort, in keeping with the established COPD diagnosis.

**Table 1:** Baseline characteristics of the clinic cohort.

Demographics			Care (n=58)	Case (n=32)	Total (N=90)
Age (years), median (IQR^a^)			64 (54-72)	71 (64-78)	68 (60-76)
Gender (males), n (%)			30 (37.5)	12 (52)	42 (47)
**Past medical history or health status**					
	**Smoking status, n (%)**				
		Current	19 (34)	11 (33)	30 (33)
		Ex-smoker	39 (59)	18 (65)	57 (63)
		Never smoker	1 (2)	2 (6)	3 (4)
	Smoking pack-years, mean (IQR)		23 (9-40)	40 (20-60)	30 (20-59)
	Body mass index (kg/m^2^), mean (SD)		32.2 (5.2)	27.26 (7.26)	28.9 (7.12)
	**Comorbidities, n (%)**				
		Cardiovascular	28 (48)	13 (41)	41 (45)
		Gastrointestinal or abdominal	23 (40)	14 (44)	37 (41)
		Previous nonthoracic malignancy	4 (8)	4 (13)	8 (9)
		Diabetes	7 (12)	7 (22)	14 (16)
	**Burden of comorbidity, n (%)**				
		Number of patients with >1 comorbid long-term condition	44 (76)	18 (56)	62 (69)
		Number of patients with >1 respiratory condition	4 (7)	6 (19)	10 (11)
**Baseline disease characteristics**					
	Forced expiratory volume in 1 s (liters), mean (IQR)		2.11 (1.72-2.63)	1.29 (0.94-1.76)	1.59 (1.03-2.14)
	Forced expiratory volume in 1 s/forced vital capacity, mean (IQR)		74.5 (69-80)	46 (37-65)	59 (44-76)
	**Medicines Research Council Dyspnea Scale scores**				
		1, n (%)	1 (3)	5 (9)	6 (7)
		2, n (%)	2 (6)	9 (15)	11 (12)
		3, n (%)	1 (3)	13 (22)	14 (16)
		4, n (%)	1 (3)	6 (10)	7 (8)
		5, n (%)	1 (3)	10 (31)	11 (12)
		Not defined, n	26	15	41
	Veterans Specific Activities Questionnaire level, mean (IQR)		5 (3-7)	3 (1-5)	3 (2-5)

^a^IQR: interquartile range.

### Diagnoses

There were 58 patients in the care cohort where a diagnosis of COPD was listed on EMIS and 32 patients (those with a suspected, but as yet established COPD diagnosis) in the case-finding cohort. Of the care cohort, 76% (44/58) remained with a diagnosis of COPD, 14% (8/58) were rediagnosed with asthma, 10% (6/58) had asthma-COPD overlap, and 1.7% (1/58) had heart failure. Of the case-finding cohort, 62% (20/32) had asthma, 9% (3/32) had COPD, 12% (4/32) had asthma-COPD overlap, and 16% (5/32) had other diagnoses (lung cancer with chronic hypersensitivity pneumonitis, reflux, or bronchiectasis).

Additional diagnoses of dysfunctional ventilation syndrome were made in 40% (23/58) of the care cohort and 44% (14/32) of the case-finding cohort using the Nijmegen questionnaire and additional clinician assessment. In one patient in the case-finding cohort, dysfunctional ventilation syndrome was the primary diagnosis. A total of 22% (13/58) of care cohort patients and 41% (13/32) of case-finding cohort patients screened positive for anxiety and depression using HADS. Obstructive sleep apnea was suspected in 9.8% (9/91) patients by using Epworth Scores or clinical suspicion, of which it was confirmed in 5 patients and disproved in 2 patients through subsequent sleep studies; the remaining patients (n=2) did not attend their follow-up clinic appointments.

Where additional comorbidity was suspected at the RCAC, patients were seen in the severe COPD assessment clinic, where access to computed tomography scanning and echocardiogram were offered in one day in addition to palliative care review, dietetics, and psychology assessments. These clinics found additional diagnoses of idiopathic pulmonary fibrosis (2 patients), bronchiectasis (6 patients), respiratory bronchiolitis interstitial lung disease (1 patient), bronchiolitis obliterans (1 patient), lung cancer with chronic hypersensitivity pneumonitis (1 patient), cavitatory lung disease with nontuberculous mycobacterial infection (1 patient), significant bullous emphysema secondary to cannabis use (1 patient), pulmonary hypertension (5 patients), left ventricular failure (4 patients), and valvular heart disease (1 patient).

### Unscheduled Health Care Usage

The impact of attendance to the MISSION COPD clinic and education sessions on unscheduled health care usage was explored by capturing each individual’s usage in the 12 months before versus 6 months after the clinic. These results show a significant reduction in GP visits and exacerbations across both cohorts (Table 2). Further sensitivity analyses were undertaken to compare changes in ED attendances and hospital admissions among those who had an episode before clinic attendance versus annualized figures after attendance. This showed a statistically significant reduction in ED visits (*P*=.01) and hospital admissions (*P*=.05).

**Table 2:** Changes in unscheduled health care usage.

Outcomes 12 months prior to 6 months after clinic attendance (with figures subsequently annualized)	Total (N=90)			Care (n=58)				Case (n=32)		
	Mean per patient at baseline	Mean per patient after	*P* value	Mean per patient at baseline	Mean per patient after	*P* value	Mean per patient at baseline		Mean per patient after	*P* value
Unscheduled general practitioner usage	3.37	1.4	<.001	3.06	1.41	<.001	3.56		1.5	<.001
Exacerbations	2.64	1.02	<.001	2.42	0.96	<.001	2.78		1.2	<.001
Out-of-hours calls	0.16	0.10	.05	0.14	0.11	.42	0.18		0.08	.70
Emergency department visits	0.12	0.5	.10	0	0	<.001	0.20		0.08	.10
Hospital admissions	0.49	0.24	.18	0.32	0	.33	0.06		0.04	.32

### Disease-Related Quality of Life Scores

SGRQ- and CAT-validated questionnaires were used to assess disease impact at baseline, 3 months, and 6 months. The SGRQ was poorly filled with only 16% (16/84) of patients returning an adequately completed series of questionnaires. Of those, 37.5% (6/16, *P*=.40) of the patients met the minimal clinically significant improvement at 3 months, with 44% (7/16, *P*=.37) showing improvement at 6 months. There was a significant improvement in the CAT scores between 3 and 6 months in the total cohort (*P*=.04) and in the care cohort (*P*=.05), but not in the case cohort (*P*=.65).

### Confidence and Patient Activation

Of the 87 responses to the PAM questionnaire at baseline, 11.5% (n=13) scored 4, indicating confidence in managing problems with their health; 34.5% (n=35) scored 3, indicating some confidence but some inability to adapt to new challenges; 31% (n=34) scored 2, suggesting they may lack basic knowledge about their health; and 23% (n=22) scored 1, suggesting that they did not know they need to play an active role in their health. An improvement in activation according to PAM occurred in 35.7% (15/42) respondents at 3 months and 42.5% (17/40) at 6 months. A greater number of patients in the care cohort than in the case cohort increased their activation levels at 6 months (48% vs 33%).

Of the 70 responders at baseline, 60% (n=42) rated themselves confident or very confident. At 3 months, 72% (21/29) of responders and at 6 months, 86% (18/21) of responders felt the MISSION clinic had increased their confidence.

### Cost

The RCAC cost £104.39 per patient. The severe COPD assessment clinic cost £410.81, making the total expenditure £515.20 per patient per severe COPD clinic.

Table 3 illustrates the changes in unscheduled health care expenditure showing a median reduction across all unscheduled health care domains. No single measure reached statistical significance. However, sensitivity analysis applied to cohorts reflecting previous usage showed statistically significant improvements in expenditure in patients with prior heavy primary care use and those with previous hospital attendance episodes.

Table 4 reflects the cost of the patient through the MISSION clinic versus current care models, indicating the economic benefit of efficiency that this streamlined model provides.

**Table 3:** Cost impact of the clinics on unscheduled health care usage comparing previous 12 months with annualized 12 months (shown as expenditure in pounds per patient).

Analysis and unscheduled care expenditure		Preclinic (£), median (IQR^a^)	Postclinic (£), median (IQR)	*P* value
**Whole cohort (N=86)**				
	Unscheduled GP^b^ visits	72.50 (0-217.50)	0 (0-145)	.10
	Out-of-hours calls	0 (0-0)	0 (0-0)	.16
	ED^c^ attendances	0 (0-0)	0 (0-0)	.36
	Hospital admissions	0 (0-0)	0 (0-0)	.48
	Total unscheduled care expenditure	145 (0-362.50)	0 (0-145)	.01
**GP use cohort (n=54)**				
	Unscheduled GP visits	217.50 (145-362.50)	0 (0-145)	<.001
	Out-of-hours calls	0 (0-0)	0 (0-0)	.03
	ED attendances	0 (0-0)	0 (0-0)	.47
	Hospital admissions	0 (0-0)	0 (0-0)	.71
	Total unscheduled care expenditure	217.50 (145-429.47)	0 (0-145)	<.001
**Hospital use cohort (n=9)**				
	Unscheduled GP visits	145 (145-290)	0 (0-145)	.35
	Out-of-hours calls	0 (0-66.97)	0 (0-0)	.08
	ED attendances	140.48 (140.48-140.48)	0 (0-0)	.01
	Hospital admissions	0 (0-2249)	0 (0-0)	.05
	Total unscheduled care expenditure	859.95 (647.98-2394.90)	0 (0-145)	.008

^a^IQR: interquartile range.

^b^GP: general practitioner.

^c^ED: emergency department.

**Table 4:** Costs of the patient through the Modern Innovative Solutions in Improving Outcomes in Chronic Obstructive Pulmonary Disease clinic versus current care models.

Cohort	Preclinic costs (£), mean	Cost of intervention (£), per patient mean	Equivalent cost of diagnostics in routine care (£), mean	Change in expenditure per patient (£), mean
Care cohort - rapid clinic only	286.77	104.39^a^	Not applicable	−98.84
Care cohort - rapid and severe clinic	440.13	544.52	798.86	−271.19
Case finding - cohort rapid only	188.31	104.39^a^	217.50^b^	−173.48
Case finding - cohort rapid and severe clinic	72.5	544.52	915.47	−487.67

^a^Cost of rapid assessment chronic obstructive pulmonary disease clinic.

^b^Cost of 3 diagnostic visits to general practitioner.

## Discussion

MISSION COPD is a novel model of care for patients with airways disease, promoting vertical integration, shared interprofessional learning, and patient education. This study suggests that MISSION COPD improves unscheduled care usage, patient activation, and health care costs.

This accompanying research project sought to assess the feasibility of this project and thus was not designed to show statistical significance. It also did not feature a control group. We need to acknowledge that there will be inherent responder bias in the returned questionnaires measuring disease-related quality of life and disease control and activation. However, health care usage was obtained from GP records and was therefore not subject to bias.

Experiences of the project have shown that diagnoses of asthma remain difficult to manage in primary care. A total of 14% of our care cohort was rediagnosed with asthma and 10% with asthma-COPD overlap. Similarly, with the case-finding cohort, the predominant diagnosis was unrecognized asthma. Given the drive to recognize the complexity of airways disease with targeting of “treatable traits” [12], there is a need to improve recognition of these patients, especially those with evidence of significant triggers or eosinophilic airway inflammation. In a similar community respiratory clinic, Hasset et al [13] found that one-third of the patients referred for likely COPD were misdiagnosed, and in our clinic, this value was one-quarter. Overall, the case-finding cohort uncovered an undiagnosed airways disease in 83% of those assessed. Of the 38 identified, 16 were receiving inhaled therapy without a formal diagnosis. Of these, two had their inhalers stopped where no treatable diagnosis was established.

The MISSION project proactively identified comorbidity through the use of validated screening questionnaires (HADS, Epworth, Nijmegen, Gastroesophageal Reflux Disease Questionnaire) followed by a medical review. A total of 41% patients screened positive for dysfunctional ventilation, although equal distribution was seen between the case and care cohorts. This suggests that, in this group, underrecognition of dysfunctional ventilation was not a significant barrier to recognition of the cause of breathlessness. Anxiety and depression was found in 29% of the patients, with a greater prevalence in the case-finding cohort. A review by Livermore et al [14] revealed that the prevalence of panic disorder in COPD is not well defined, with evidence showing a range of 6%-67%; however, there are consistent reports that the presence of anxiety symptoms with COPD predicts an increased duration and frequency of hospital admission. A recent report by the Kings Fund [15] reveals that, independent of the severity of COPD, comorbid anxiety and depression worsen health outcomes and breathlessness with a greater impact in those of lower socioeconomic status. Furthermore, poor mental health will result in lower adherence to using prescribed medications. Both of the abovementioned works highlight the importance of screening and treatment efforts, but suggest that, even with proactive screening, we may have underrecognized cases and further work may be needed.

The additional comorbidities found at the severe clinic highlight the presence of a complex group of patients where unrecognized comorbidity may be a significant contributor to the burden of symptoms and subsequently the drive to health care consumption. These patients were sought proactively, suggesting that it may have been many months, if not years, before these conditions were recognized and addressed.

Unscheduled care usage, defined as unplanned GP visits, calls to the GP out-of-hours service, or hospital attendances were reduced for both sectors. However, the biggest impact was in the reduction in exacerbations and GP attendances. The numbers of hospital attendances and admissions in the 12 months before the MISSION clinic were low in this cohort, but it is noteworthy that one ED visit costs almost twice that of an unscheduled GP visit; therefore, fewer reductions are needed to lead to savings in the health economy. A review by Roland and Abel [16] found that the best strategy of targeting patients at risk of admission yields greater savings than those already in a pattern of repeat admission, supporting the proactive case identification favored by MISSION. Sensitivity analyses support the significant benefits in reduction of hospital attendances or admissions, if patients are prioritized for this clinic on the basis of such episodes.

Sustained improvements were seen in the SGRQ, although these were more evident among the case cohort. This demonstrates value in case finding, where addressing symptom drivers with the patient can allow for effective treatment and self-management. Significant reductions were seen in the CAT scores at 6 months, supporting this model as an effective method for achieving disease control in addition to the quality of life.

Patient activation and confidence in self-management were shown to improve and to be sustainable. Over the longer term, this has the potential to reduce cost to the health system, improve patient empowerment, and ultimately make them active participants in their health care [17].

MISSION COPD sought to deliver gold-standard comprehensive integrated COPD care that met the needs of this complex group of patients. This goal is not new, but a recent Cochrane review [4] did not find evidence of another project that met all of their recommended inclusions for a gold-standard service. Through this project, we have shown that the model is feasible and cost saving. More importantly, it delivered improvements in disease control, quality of life, and confidence of patients. The MISSION team will now test this model on larger scales to demonstrate the replicability of these results.

Cost savings have been demonstrated across all domains. No single measure reached statistical significance when considering the whole cohort, although it is noteworthy that the study was designed as an improvement program; therefore, sample sizes were defined by the delivery design and not powered for significance. When cost measures were compared for patients who had attended secondary care through ED or were admitted for the following episodes before the clinic, a statistically significant cost reduction was seen. Cost savings have been calculated using each patient as their own control, which we believe has rigor, as no other national or local initiative was delivered to this population. Thus, there were no other possible influences on participant outcomes. The calculation of equivalent diagnostic and follow-up visits to primary or secondary care providers also demonstrate the cost efficiencies that can be made by delivering these one-stop or two-stop clinics rather than the multi-visit, multisector model that currently exists. We believe this shows health economic benefits, both in its current manifestation and in case the inclusion criteria were narrowed further to include only those presenting to or admitted to the hospital.

The MISSION COPD pilot was a small-scale project testing the feasibility and acceptability of the model. To demonstrate the significant impact on delivery, particularly in realizing pounds-in-pockets savings, the model needs to be tested to scale. Outcomes were only measured up to 6 months. To ensure that improvements are sustainable beyond the initial follow-up period, further 12-month data should also be sought in scaled tests to allow for greater generalizability.

## Abbreviations

CAT: COPD assessment test
COPD: chronic obstructive pulmonary disease
ED: emergency department
GP: general practitioner
HADS: Hospital Anxiety and Depression Score
IQR: interquartile range
MISSION COPD: Modern Innovative Solutions in Improving Outcomes in Chronic Obstructive Pulmonary Disease
PAM: patient activation measure
RCAC: rapid COPD assessment clinic
SGRQ: St George’s Respiratory Questionnaire
